# Overview of Neural Tube Defects: Gene–Environment Interactions, Preventative Approaches and Future Perspectives

**DOI:** 10.3390/biomedicines10050965

**Published:** 2022-04-21

**Authors:** Jasmina Isaković, Iva Šimunić, Denis Jagečić, Valentina Hribljan, Dinko Mitrečić

**Affiliations:** 1 Omnion Research International Ltd., Heinzelova 4, 10000 Zagreb, Croatia; 2 Department of Histology and Embryology, University of Zagreb School of Medicine, 10000 Zagreb, Croatia; denis.jagecic@mef.hr (D.J.); valentina.hribljan@mef.hr (V.H.); dinko.mitrecic@mef.hr (D.M.); 3 Laboratory for Stem Cells, Croatian Institute for Brain Research, University of Zagreb School of Medicine, 10000 Zagreb, Croatia; iva.simunic25@gmail.com

**Keywords:** neural tube development, neural tube defects, congenital malformations, spina bifida, anencephaly

## Abstract

Neural tube defects (NTDs) are the second most common congenital malformations of humans, characterized by impaired development of the central nervous system. Even though the etiology of most birth defects remains undetermined, genetic and environmental risk factors in the background of NTDs have been identified and extensively reported. On top of genetic and nutritional risks which include mutations in both coding and non-coding regions and maternal folate status, respectively, recent years have seen a rise in the identification of a variety of teratogens that could be implicated in NTD development. These include polycyclic aromatic hydrocarbons, arsenic, pesticides, maternal hyperthermia and antibiotics as well as pain and seizure medication. With an increase in understanding of teratogens leading to NTD formation, preventative and treatment approaches have witnessed great advances throughout the years. While the most common preventative approach includes folic acid food fortification as well as suggested inositol supplementation, treatment and management approaches differ greatly depending on the developmental stage and the site of the lesion and include prenatal surgery, stem cell transplantation and postnatal surgery. Because NTDs still represent a large health and financial burden for the patient and society as a whole, it is crucial to investigate potential risk factors and develop novel approaches in order to fully prevent this category of disorders.

## 1. Introduction

Congenital malformations entail single or multiple defects in the morphogenesis of organs that arise during embryogenesis and fetal development due to genetic mutations or maternal exposure to environmental factors, including infection. Together with neonatal disorders, congenital birth defects represent some of the leading causes of global disability-adjusted life years (DALYs) for both sexes, combined for all ages.

Primary congenital malformations arise from intrinsic errors within the developmental process and are of genetic origins. Those of secondary nature occur when environmental factors disrupt an otherwise normal developmental process during intrauterine life. These include any substances introduced into the organism (drugs, alcohol, smoking, etc.) or directly influencing exogenous factors (external temperature, environmental pollutants, etc.). These may cross the placenta and reach the fetus, inducing changes in the embryo–fetal development [1]. Estimates from the World Health Organization determine that 6% of infants worldwide present with congenital anomalies [2].

Neural tube defects are congenital malformations which arise during the abnormal embryonic development of the central nervous system. They include spina bifida, encephalocele and anencephaly, on top of many others. While spina bifida leads to impairment or loss of motor, sensory and autonomic function through damaging the spinal cord and nerves, encephalocele entails cerebral herniation, resulting in a variety of cognitive impairments [3]. On top of these functional impairments, most NTD patients also exhibit cosmetic concerns that might contribute to their morbidity as well as their psychological and financial conditions, greatly diminishing their wellbeing.

In order to combat the rising toll these congenital malformations have on infant survival and their quality of life, elaborate folic acid fortification programs have been introduced in many regions, including set requirements for food products such as wheat and maize flour [3]. In spite of elaborate folic acid fortification programs, many nations worldwide still experience disproportionately large numbers of NTDs and NTD-related deaths. This is largely due to the economic, health and social disparities present in those regions. As seen in the Global Burden of Disease data, the majority of deaths caused by NTDs (85%) occur in nations within the lowest GDP quartile [3], contributed to by limited access to neurosurgical care as well as inefficient prevention and education programs.

Therefore, even though we are witnessing a rise in improved diagnostic and therapeutic tools for prevention, management and treatment of congenital malformations, they still represent a major issue in the contemporary world.

## 2. Neural Tube Formation and Defects

### 2.1. Neural Tube Development

The third week of embryonic development in humans is marked with gastrulation, a process in which the three germ layers are formed: ectoderm, mesoderm and endoderm. The ectoderm further thickens in response to molecules which underly the notochord secretes, giving rise to the neural plate.

While the neural plate is the basis for primary neurulation, i.e., for the process of forming the neural tube from the neural plate, in amphibians, birds and mammals, the caudal portion of the neural tube develops directly by differentiation of the mesenchymal stem cells which represent the remnant of the primitive streak [4,5]. The process of the elevation and fusion of the opposing parts during formation of the (primary) neural tube is controlled by many signaling pathways, which can be, in general, described as either those coming from the notochord or those coming from the surrounding—for example, somatic—tissue. Signals coming from the notochord—such as Shh—have a strong ventralizing influence, i.e., they trigger many secondary genes which are then involved in the differentiation of the ventral specificity of the neural tube (e.g., *OLIG2*, *NKX2-2*). At the same time, the secretion of BMPs by the surface ectoderm and underlying mesoderm influences the differentiation of the dorsal portions in the closing of the neural tube.

Interestingly, the closure of the neural tube is species specific, and it differs in the number and position of sites which initiate the zippering. For example, while in birds, only two starting positions for the zippering exist (one cranial and one caudal), mammalians possess several closing regions in which closing occurs at the same time. While a pig possesses a huge region in the future thoracic portion of the body in which neural tissue first apposes and then fuses, humans possess at least four different regions in which zipper-like closing occurs at the same time [6]. Failure in the formation of the described processes results in different abnormalities called neural tube defects (NTDs) [7].

### 2.2. Neural Tube Defects (NTDs)

**Figure 1:**
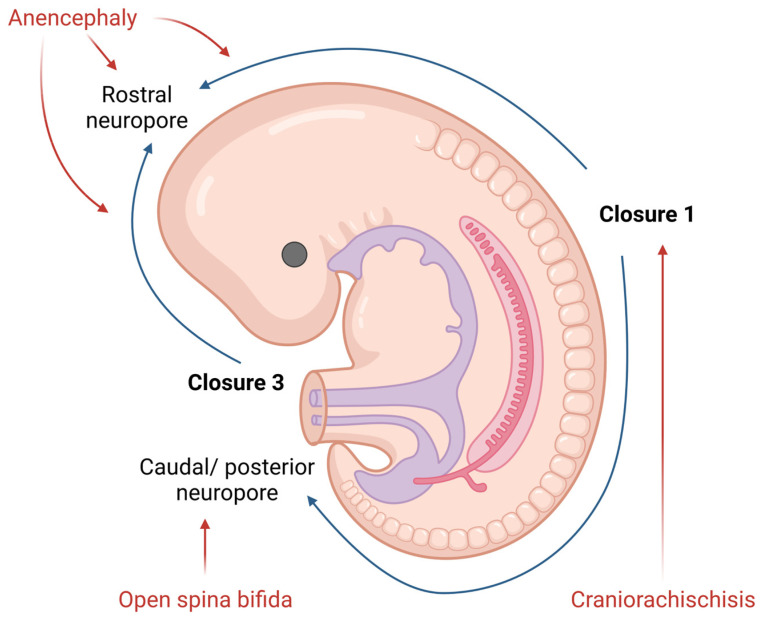
Diagram of neural tube closure and the origin of open NTDs in human embryos. Created with www.BioRender.com (accessed on 26 March 2022).

NTDs are one of the most common congenital malformations in humans. They form during the embryonic development and affect life quality from birth. The pathogenesis of NTDs has not yet been fully clarified, but nowadays, we know both genetic and environmental factors contribute to, or have an important role in, this malformation [8]. The presentation of NTDs is extremely variable, mostly depending on the localization of the lesion. Therefore, NTDs could be divided into open (Figure 1) and closed defects, depending on the exposure of the brain and/or spinal cord.

Open NTDs (ONTDs) such as craniorachischisis, anencephaly and myelomeningocele appear when there is a defect in the skull or vertebrae (Figure 1). Because the neural tissue is then exposed to the inherent toxicity of the amniotic fluid, this, consequentially, leads to the degradation. The diagnosis of such is usually set by an ultrasound. Despite an ultrasound, screening for the increase in the alpha-fetoprotein (AFP) concentration in the maternal serum became popular in NTD diagnostics as well [9].

The most severe defect, which includes both the cranial and spinal part of the neural tube, is craniorachischisis. This defect results in anencephaly and open spina bifida with an absence of the cranial vault and various defects of the vertebrae that can be seen on an ultrasound. Such condition is lethal, and no cure or surgical approach is known; therefore, the death of a newborn is unavoidable.

Anencephaly is a condition wherein the defect only affects the cranial part of the neural tube. Here, an absence of the forebrain and the skull can be noticed. Despite the anomalies of the calvarium [10], the facial bones appear normal. Furthermore, reported facial abnormalities include a flattened nasal bridge, low-set ears and eyes that seem to protrude [11]. Except for the aforementioned morphological features which could be seen on a routine ultrasound, the area of the cerebrovasculosa (residual cerebral tissue) and polyhydramnios (due to the lack of the swallowing reflex) could be detected. Like craniorachischisis, anencephaly also represents a lethal condition.

The failing posterior neural tube formation leads to myelomeningocele, a condition characterized by a protruded meningeal sack with neural placodes. However, when only the neural tissue is detectable, without the cystic sack, we talk about myelocele. Chiari malformation type II is usually associated with myelomeningocele and myelocele due to the traction of the brain stem throughout the opening in the vertebrae. The diagnosis of these is based on the detection of the “lemon” or “banana” indirect signs on a routine ultrasound checkup [12]. These NTDs present themselves as reduced leg movement in utero, due to the damage of the spinal cord, and various degrees of neurological impairment post birth. Even though these conditions entail debilitating impairments, newborn survival is high.

On the other hand, closed neural tube defects include the malformation of the fat, bone or membranes in the spinal column. The most common and powerful diagnostic tool used to detect these abnormalities is an ultrasound.

Encephalocele appears when a sack-like protrusion of the brain and/or meninges herniates through an opening in the skull. According to the tissue present in a herniated sack, cephaloceles could be classified as encephalomeningocystocele (herniated meninges, neural tissue and ventricles), encephalomeningocele (herniated meninges and neural tissue) and meningocele (herniated meninges). Depending on the location of the lesion, encephalocele are divided into anterior and occipital. Anterior encephalocele is subdivided into three more categories, frontal, sincipital and basal, while occipital is connected to Chiari malformation III. These abnormalities can be visualized using an ultrasound, where they present themselves as a sack protruding through a bone defect. Surgical correction of these is required after birth.

When compared with encephalocele, meningocele entails herniated meninges protruding through a vertebral arch defect, earning it the name of closed spine bifida. The herniated mass consisting of the dura and arachnoid is covered by skin which has atrophic epidermis without skin appendages. During the ultrasound examination, a cystic mass with spinal dysraphism is detected. The differences between meningocele and myelomeningocele are visible in cranial anatomy and the septate herniated mass observed in meningocele.

The aforementioned vertebral arch defect is also present in spina bifida occulta; however, no herniation or cystic masses are present. As such, the signs of spina bifida occulta are not as clear as the ones characterizing other NTDs. Therefore, it is important to look for cutaneous markers, including hair tufts, nevi, capillary hemangiomas, depigmentation regions or subcutaneous lipomas [13,14]. Because of this, spina bifida occulta is usually not recognized during pregnancy or in the perinatal period. Nevertheless, the most common type of spina bifida occulta with intradural lipoma could be detected in utero because lipomas and meningoceles have a similar appearance. The diagnosis of such NTDs is made easier in the postnatal period where a high-resolution ultrasound can be used. However, its sensitivity is affected by the presence of subcutaneous masses, impacting the diagnostic procedure quality. Even though the most precise diagnosis is made using magnetic resonance imaging, it is rarely used as the primary approach due to the high cost of this procedure. Because of this, the diagnosis of spina bifida occulta is usually made later in life because there is no disability present early on, and the first symptoms appear after the damage and/or traction of the cord.

## 3. Etiology and Pathogenesis of Neural Tube Defects (NTDs)

### 3.1. Nutritional Risk Factors

Maternal folate deficiency before conception and during the first trimester of pregnancy is one of the main risk factors for neural tube defects. Folate (vitamin B9) is used as a generic term for a family of chemically and functionally related compounds, including folic acid, dihydrofolate (DHF), tetrahydrofolate (THF), 5, 10-methylenetetrahydrofolate (5, 10-MTHF) and 5-methyltetrahydrofolate (5-MTHF). [15]. The folates found in food are mainly in a polyglutamate form, while folic acid, the synthetic form of folate used in many supplements, is in a monoglutamate form. Although their metabolism is slightly different in some steps, most dietary folates and folic acid that are added to the diet share a common metabolic fate, as they are metabolized to L-5-methyltetrahydrofolate (L-5-MTHF) during their passage across the intestinal mucosa [15]. Folate coenzymes are required for the synthesis of some of the nucleic acid building blocks (thymidylate and purines), a synthesis of methionine from homocysteine, and the interconversion of serine and glycine [15]. 

Folate status might influence the methylation of DNA and histone modifications, thus altering the expression of some genes involved in neurodevelopment. For example, differential methylation in relation to folate from maternal plasma was found for some of the genes implicated in NTDs analyzed from cord blood [16]. In addition, data from clinical samples revealed the presence of aberrant DNA methylation in a GNAS imprinting cluster in NTD samples with low folate concentrations [17]. Folate deficiency conditions increased histone H2A monoubiquitination (H2AK119ub1) which downregulated expression of the neural tube closure-related genes *Cdx2*, *Nes*, *Pax6* and *Gata4* in mouse embryonic stem cells [18]. Although some of the mechanisms are being elucidated, further research is needed for a better understanding of the mechanisms behind the detrimental effect of folate deficiencies on the development of NTDs.

### 3.2. Genetic Risk Factors

Animal models are very important for a better understanding of neural tube development as they can point to several candidate processes for study in the human NTDs etiology [19]. There are 240 mouse mutants and strains with neural tube defects which identify the genes needed for embryonic neural tube closure (NTC) [19]. It is important to mention that having a mutation does not have to mean a certain development of NTDs because, in most cases, environmental factors play a crucial role alongside genes. For example, the prevention of NTDs by maternal folate supplementation has been tested in 13 mutants and, subsequently, led to a reduction in NTD frequency in six diverse mutants [19]. Examples of gene mutations that cause NTDs in mice, and also implicated in the minority of NTD cases observed in humans, are genes for low-density lipoprotein receptor-related protein 6 (*Lrp6*) and paired box 3 protein (*Pax3*) [20,21]. In some cases, an NTD can be secondary, caused by the malformation of other structures, such as the notochord [5].

Alongside animal models, important insights into genetic risk factors for NTDs are obtained using next-generation sequencing. This can include whole-genome sequencing (WGS), whole-exome sequencing (WES) or targeted panel sequencing. Using WES, Singh et al. identified a homozygous missense mutation in the *TRIM36* gene as the cause of autosomal recessive anencephaly in an Indian family [22]. *TRIM36* is expressed in the developing human brain, suggesting a role in neurogenesis [22]. WES also revealed that de novo damaging variants could be the main culprit for the majority of anencephalic cases [23]. The targeted exome sequencing of 191 NTD candidate genes of 90 patients with cranial NTDs identified 397 rare variants, 21 of which were previously unreported and predicted to be damaging [24]. Mutations can also be found in non-coding regions. For that reason, whole-genome sequencing (WGS) is an important tool for discovering potential mutations contributing to NTDs risk, as shown by Aguiar-Pulido et al., who found mutations in the regulatory regions of several transcription factors critical to embryonic development [25].

### 3.3. Gene–Environment Interactions

#### 3.3.1. Polycyclic Aromatic Hydrocarbons

Polycyclic Aromatic Hydrocarbons (PAHs) are environmental pollutants shown to have an adverse effect on human health, one of which is an increased risk of NTDs. Most PAHs are generated from anthropogenic activities, primarily during the incomplete combustion of organic materials such as coal, oil, petrol and wood [26]. A study conducted in the rural population in Shanxi Province (China) showed that women with any exposure at all to indoor air pollution from coal combustion (IAPCC) had a 60% increased risk of having a child with an NTD, compared to women with no IAPCC exposure [27]. Additionally, a study by Langlois et al. suggests that maternal occupational exposure to PAHs may be associated with an increased risk of spina bifida in offspring among women who are normal weight or underweight [28]. One study showed that PAH concentrations in the placenta of cases with NTDs were significantly higher than in the controls [29]. An increased concentration of PAHs in maternal serum was also associated with an elevated risk for NTDs [30]. Although the exact mechanisms underlying the association between PAHs and NTDs remain largely unknown, decreased global DNA hydromethylation could be one of them [31]. Considering the PAHs negative influence on overall human health, increased risk of NTDs included, it is important that remediation approaches for these contaminants are being developed [26,32].

#### 3.3.2. Arsenic

Out of the 275 most hazardous environmental substances, arsenic presents potentially one of the greatest dangers to human health [33]. The global arsenic level is influenced by many different factors and activities, such as metalworking industries, pesticide production, coal combustion, etc. Because of this, inorganic arsenic is detected in contaminated water, soil and air that can, by consumption of polluted food and water, easily enter the mammalian system. In fact, 95% of the average arsenic intake among Europeans comes from contaminated food.

Arsenic has well-known teratogenic and toxic properties and is one of the major risk factors for developing NTDs [33,34,35]. It leads to direct cytotoxic and teratogenic effect by the production of reactive oxygen species (ROS) and inhibition of antioxidative enzymes. It can also affect cells on the level of DNA, where it inhibits DNA methylation and DNA repair mechanisms. The cellular metabolism is disrupted by the induction of mitochondrial stress, inhibition of oxidative phosphorylation and alterations in the glucose levels. On the other hand, the teratogenic properties of arsenic are connected with the damage of microvascular placental structures, thus disrupting the transport of molecules and nutrition [34,35,36,37]. Because arsenic has the ability to accumulate in the neuroepithelial tissue during embryogenesis, gene mutations and epigenetic changes from arsenic poisoning directly induce NTD formation in animal and human studies [35,36]. GWAS (genome-wide association studies) identified 14 SNPs (single nucleotide polymorphism) expressed in NTDs after arsenic poisoning [35].

DNA methylation is one of the most important and well-known developmental processes in the human body that is heavily affected by arsenic exposure. Studies have shown that arsenic exposure decreases the activity of SAM (S adenosyl methionine) as well as DNM1 and 3b (DNA methyltransferase) by 34.1 and 60.2%, respectively, thus inhibiting DNA methylation. In animal studies where a confirmed disruption in DNA methylation was quantified, 33.3 ± 9.4% of embryos with arsenic-induced spina bifida were observed [36]. Another study conducted on 48 stillborns with NTDs and 49 aborted controls in China clearly showed a statistically significant decrease in the methylation pattern of genomic DNA and LINE (long interspersed nuclear element), when compared to the control group [38].

As it was mentioned previously, a number of NTDs can be reduced by regular folate intake during pregnancy. Because folate usually enters the body through folate receptor-1 (FolR1), its activity is largely dependent on arsenic exposure. Research performed by Wlodarczyk et al. reported a 64% occurrence of exenchephaly on *FolR1* mice nullizygous. Furthermore, it has also been shown that folate and arsenic interact with ATP-driven efflux pumps which results in high folate efflux and the reduction in arsenic levels in blood [34]. For example, the acid supplementation trial in Bangladesh included participants with low plasma levels of folate who randomly received folate or placebo. Results showed that the folic acid group excreted significantly more urinary arsenic which resulted in lower blood arsenic levels [35].

Additionally, a case study which included 49 mothers and their newborns showed high correlation between placental and environmental arsenic levels. They also investigated the combined effect of smoking and living near the smelter which decreased the level of antioxidant enzyme glutathione and increased the levels of lipid peroxides in maternal blood and placenta, indicating elevated oxidative damage [37]. Another case–control study performed in Bangladesh, where between 35 and 77 million people have been exposed to high arsenic levels through contaminated water, connected arsenic exposure to NTD development. In this study, the estimated levels of arsenic in the water exceeded the World Health Organization drinking water guidelines by 100 times (1000 ug/L compared to recommended 10 ug/L) [35]. During this time, India and some parts of China also revealed higher numbers of NTD cases. Because these regions mostly rely on rice and groundwater consumption, there was a high likelihood of arsenic poisoning [33].

#### 3.3.3. Pesticides

The increase in the global population observed during the last few decades demanded the development of new food production strategies. These strategies mostly relied on pesticide implementation, without any knowledge of its general risk or effects on the environment and overall health. As such, the following years have seen a rise in publications emphasizing serious medical side effects and the dangers of such overwhelming pesticide use [39,40]. Because pesticides are non-biodegradable, they can easily enter the human body through contaminated food and water. This is followed by pesticide accumulation in adipose tissue and placenta. Through that, they cause severe reproductive and developmental disorders with teratogenic and cancerogenic potential, as well as lead to immunological and neurological dysfunction [39,41,42]. Because of this, we have witnessed a proposed total ban of several different pesticides at the Stockholm Convention on Persistent Organic Pollutants in 2001, which was further extended in 2009 (dieldrin, endosulfan, α-HCH, β-HCH…) [39].

Many research studies and case reports connected pesticide exposure with the development of congenital disorders, such as NTDs [39,40,41,43,44]. Levels of these pesticides and, consequently, their role in NTD development can be measured directly and indirectly. Indirect methods include occupational exposure and residential proximity to agricultural centers or cultivated fields, while direct methods include the measurement of different pesticides from the placenta or maternal and newborns’ blood [39,40,45]. A study performed near Hanford in Washington, a location notorious for the widespread use of herbicides, recorded 23.000 births and revealed an increased rate of NTDs [46]. A case–control study carried out in California concluded that women with higher pesticide exposure have an elevated risk of NTD pregnancies [47]. Another case–control study was performed in India, this time on the blood samples of 35 mothers and their children with NTDs, as well as 35 mothers with children without congenital disorders. It measured the blood levels of a few different organochlorines, and the results showed a significant increase in certain pesticides in both the blood of the mothers (11.3× increase in Dichlorodiphenyldichloroethylene) and NTD neonates (3.6× increase in endosulfan), when compared to the control group [39]. Additionally, a study performed on a case group of 184 and a control group of 225 Mexican American women revealed a 2× higher chance of NTD-affected pregnancies in the case group after home usage of pesticides or living within 0.25 miles of agricultural fields [40]. The odds ratio (OR) increased with every new maternal pesticide exposure. Nevertheless, the levels of hydrocarbon insecticides and methyl parathion in the blood and urine samples were below delectable levels for the majority of mothers. Because this study was performed retrogradely, some of the results might have been impacted by differential recall [40]. 

On the other hand, a population-based case–control study conducted in the Mexican American population, and including the surveillance of 21 hospitals, 39 birthing centers, 4 genetic clinics, 74 ultrasound centers, 4 abortion clinics and 150 midwives of the Brownsville cluster, indicated that case mothers in that specific area had little to no risks of NTDs after pesticide exposure [45]. In spite of that, it was crucial to observe other comorbidities or confounding factors which could lead to an increased risk of NTDs after pesticide exposure, some of which included B12 and folate deficiency. The examination of B12 serum levels indicated a strong, consistent relation with NTD risk post-pesticide exposure [48]. On the other hand, no difference was observed between the serum or RBC folate levels in case-mothers and control-mothers (serum folate ng/mL, 11.2 vs. 11.4; red blood cell (RBC) folate ng/mL, 339 vs. 360), indicating no clear relation between folate deficiency and increased risk [48]. Still, the same study concluded that women in the lowest quintile have a slightly increased risk (OR = 1.3, 95% CI: 0.6, 2.7) compared with those in the highest. From that, a conclusion was derived that the most important risk factors, or comorbidities, associated with NTD development following pesticide exposure were low levels of vitamin B12 and—to a much lesser extent and only in a certain subset of the population—decreased folate intake [45]. Additionally, because this study was performed in a region with a longstanding tradition of corn consumption, the risk for NTD development was higher than the average due to ongoing exposure to maize contaminated with mycotoxicant fumonisin.

A systemic review which included all major scientific databases concluded that some third world countries, like many in Africa, where agricultural pesticides are commonly used, have a 3× higher rate of NTD occurrence in newborns, when compared with developed countries. Moreover, it has been noted that some parts of Central and Southern Africa, where folic acid fortification is regularly and increasingly being performed, had a decreased number of NTDs [42]. Another case report study performed on 81 cases and 162 control women in Ethiopia, which was based on a systematic questionnaire written in accordance with WHO guidelines, determined the adjusted odds ratio (AOR) of 5.3, indicating a much higher incidence of NTD newborns following maternal pesticide exposure [41]. Additionally, some studies, such as the one conducted in England and Wales, showed that gardeners and groundsmen have a higher paternal risk of having offspring with NTD-related disorders [49].

#### 3.3.4. Maternal Hyperthermia

Many studies reported the influence of hyperthermia on the development of congenital diseases, such as NTDs. Because the brain is extremely sensitive to hyperthermia during the early gestational period, animal studies and case reports indicated its teratogenic and mutagenic effects [50,51,52,53,54,55,56]. As such, an increase in maternal core temperature by the effect of some internal (viruses) or external sources (sauna, warm bath, hot tub and electric blanket) to 40 °C is considered potentially damaging for the fetus and can, as such, cause developmental and genetic abnormalities [51,52].

In general, the development of NTDs is closely related with the degree of hyperthermic damage as well as the overall exposure time. Guidelines proposed by Harvey et al. limited the use of hot tubs for pregnant women to 39 °C for 15 min and less than 10 min at 40 °C [57]. On a cellular level, elevated body temperature disrupts the mitotic processes and causes changes in gene expression [52,53,56,58], the most important of which is the gap junction α-1 gene (*GJA1*) which encodes for the gap junction protein connexin 43 (Cx43). Cx43 plays an essential role in neural tube development by creating gap junctions between cells. What is interesting to note here is that an animal study, performed on golden hamsters, indicated a significant overexpression of Cx43 mRNA in the neural tube in the heat-threated group when compared to the control, giving rise to a direct argument connecting the expression of Cx43 with NTDs [52]. Additional animal studies also revealed that the strain differences observed in responses to hyperthermic exposure are mostly likely caused by single fetal genetic locus and maternal effect [53].

Similarly, some viruses, such as influenza, can directly affect the fetus by increasing the maternal body temperature. This leads to the subsequent production of toxic metabolites that can easily pass directly through the placenta or indirectly through antiviral and antipyretic medications. First-trimester influenza exposure poses an increased risk of NTD development (OR = 3.3). Interestingly, when the obtained results were adjusted for the use of antipyretic and antiviral treatments, a slight decrease in the trend was observed. Nevertheless, the results still remained significant [54]. Nine case report studies, which included 1601 newborns with NTDs and 5149 controls, demonstrated a clearly elevated OR of 1.93 after maternal exposure to hyperthermia [50]. Similar results were shown in a study performed in California, where the influence of maternal fever or febrile illness was studied in 538 NTD cases and 539 controls [59]. The results have shown an OR of 1.91 for fever and an OR of 2.02 for febrile illness. Additionally, a comparative study performed by Milunsky et al., which included 23,491 women, associated their exposure to various sources of hyperthermia, such as a hot tub, sauna or fever, with the development of NTDs [60]. Maternal use of a hot tub during the first trimester showed an increased risk ratio (RR) of 2.8 for a sauna, an RR of 1.8 for a fever and an RR of 1.2 for the use of an electric blanket. As such, when two of these types of heat exposure are combined, or used consecutively, the RR for NTD development increases from 1.9 to 6.2. Similar results were also shown by Salih et al. who concluded that a combination of sauna, febrile illness and hot tub use increases the risk of NTDs development by 6× [55]. Another comparative study performed in Texas by Suarez et al., involving 175 NTD positive cases and 221 controls, noticed that first-trimester maternal exposure to hyperthermia increases the RR to 3.6 [61]. Many more meta-analyses, such as the one by Morreti et al., which includes case studies from 1966 to 2003, totaling in 1719 NTD cases and 37,898 controls, connected NTD development with maternal hyperthermia, with an OR of 1.92 [50]. As indicative as these results are, it is also important to note that meta-analyses and case report studies do have certain limitations, one of the most important of which is adjusting for the confounding variable which sometimes is not applied [54]. Additionally, because NTDs develop during the first trimester, retrospective questioning is subjected to recall bias [56].

Following concerns raised regarding COVID-19 vaccination during pregnancy as it could lead to maternal hyperthermia and, subsequently, congenital malformations, Blakeway et al. performed a cohort study of pregnant women who gave birth at St George’s University Hospitals National Health Service Foundation Trust in London, United Kingdom, between March 2020 and July 2021 [62]. The study included 1328 pregnant women of whom 140 received at least 1 dose of the COVID-19 vaccine before giving birth and 1188 women who did not. After collecting data on COVID-19 vaccination uptake and type, gestational age at vaccination and maternal characteristics, additional data were collected on perinatal outcomes, including fetal and congenital abnormalities. In a propensity score–matched cohort, no significant difference in rates of adverse pregnancy outcomes in vaccinated and unvaccinated pregnant women was observed (*p* > 0.05 for all): stillbirth (0.0 vs. 0.2%), fetal abnormalities (2.2 vs. 2.5%) [62]. The three cases of fetal abnormalities reported in women who received the COVID-19 vaccine included spina bifida, ventriculomegaly and hydronephrosis, with the spina bifida case diagnosed before the pregnant woman received the first dose of the vaccine. Both cases of hydronephrosis and ventriculomegaly presented with no associated abnormalities at birth [62]. Contrasted to other studies which compare the outcomes of vaccinated pregnant women with historic background rates [63], or do not include any controls at all [64], limiting the conclusions drawn within them, Blakeway et al. included a contemporaneous control group. Furthermore, a study performed by Ruderman et al. also found no association between COVID-19 vaccination during early pregnancy and congenital fetal abnormalities [65]. This cohort study was performed on 3156 women, 2662 of which received at least one dose of the vaccine, at a quaternary medical center in Chicago, Illinois. Congenital anomalies identified on ultrasonography were reported in 27 of 534 unvaccinated people (5.1%) and 109 of 2622 people who received at least 1 dose of the vaccine (4.2%) (*p* = 0.35). As such, these studies have contributed to the growing body of evidence suggesting that COVID-19 vaccination in pregnancy does not alter perinatal outcomes [66,67,68,69,70,71,72,73]. Nevertheless, additional studies evaluating the long-term effects of maternal hyperthermia post-COVID-19 infection or inoculation are still lacking but are to be expected in the upcoming years.

#### 3.3.5. Antibiotics, Seizure and Pain Medication

Nowadays, it is known that drugs and other medication could act as teratogens, substances which cause physical or functional defects in the human embryo or fetus. Therefore, it is crucial to research which drugs and other substances fall under this category and, even more importantly, which of these are being taken as chronic therapy during pregnancy. As such, to ensure the health of the pregnant woman and her child, the FDA has divided drugs into five categories, depending on their teratogenous effect [74].

Antibiotics are often used in pregnant women to fight off different infections, such as the common urinary tract infection (UTI) or any other type of bacterial infections. For example, erythromycins are usually used to fight Gram-negative infections but also infections caused by mycoplasma, chlamydia, rickettsia or treponema. In other words, they could be used to treat respiratory tract infections, UTIs, skin infections and syphilis as well as for replacements for penicillin given to allergic patients. Despite all the positive effects of antibiotics, multiple studies have shown that these drugs could cause anencephaly, one of the most prominent being a large population-based, multisite, case-control study performed by Crider et al. [75]. Furthermore, sulfonamides—the antibiotics used for treating both Gram-positive and Gram-negative infections, toxoplasmosis, plasmodium and pneumocystis—have also been proven to possess teratogenic properties, causing a variety of neurological disorders, one of which is anencephaly.

Seizure medication is usually administered in a sequence within a chronic therapy regime. Therefore, it is especially important to take a detailed medication history in the case of an ongoing or planned pregnancy as some anti-epileptics are demonstrated teratogens. Even though there are many newly discovered anti-epileptics that have a more selective effect, combined with less adverse effects, valproic acid and carbamazepine are still often used. On top of this, despite their anti-epileptic effect, common anti-epileptics can also be used to treat bipolar affective disorder, schizophrenia and pain disorders. The most common NTD associated with the aforementioned anti-epileptics is spina bifida [76].

Pain medications are the most commonly used type of drugs. They entail paracetamol, non-steroid anti-inflammatory drugs (NSAIDs) and opioids. The type of drug used for treatment is determined for the specific pain intensity examined with a visual analogue scale (VAS). In 2017, a CDC study showed that the concurrent use of NSAIDs and opioid drugs for pain relief was related to higher incidences of spina bifida when compared to singular pain medication use [77]. Two additional studies have also confirmed the same harmful effect of opioids [78,79].

## 4. Prevention of NTDs

### 4.1. Folic Acid

Large clinical trials performed in 1991 clearly showed that periconceptional uptake of 0.4 mg of folic acid (FA) reduces the risk of neural tube defects (NTD) by 72% [80]. However, a recent population-based study performed in the United States which covered the years 1999–2011 [81] suggests that, even though it is still significant, the reduction in NTD rates by folic acid is more modest than previously predicted [82]. The factors that could have helped contribute to this difference include lower adherence to folic acid supplementation, a gradual increase in the number of annual live births in the US during the post-fortification period and data variations caused by differences in the surveillance methodology [81].

Nevertheless, one of the most obvious proofs of the beneficial effect of FA can be seen in the comparison of prevalence between Canada and the EU. Canada, like many other countries, started to implement obligatory supplementation of food with folic acid in 1998 and, in only five years, reduced the total prevalence of NTDs by almost 50% [83]. This includes an equally successful decrease in the prevalence of spina bifida, anencephaly and encephalocele, while malformations such as iniencephaly have completely disappeared. During the same time period, the prevalence of NTDs did not greatly change in the EU, which still does not regulate obligatory fortification of food with FA. Although FA reduces the prevalence of NTDs, the mechanism of its action is still not completely clear. However, research based on murine models (e.g., *Pax3*-knockout mice) that spontaneously develop NTDs, which can then be corrected through FA supplementation, made a huge progress in understanding the positive effects of FA. Thus, Sudiawala et al. showed that deficiency of *Pax3* in the dorsal neuroepithelium leads to reduced cell proliferation and premature cell differentiation [84]. Here, FA promotes progress through the S-phase of the cell cycle, thus increasing the rate of cell proliferation and preventing premature differentiation, resulting in a significantly reduced prevalence of NTDs.

On the other hand, some NTDs require additional efforts to be completely understood and prevented. Thus, while in the majority of animal models of NTDs (e.g., *Pax3*, *Mrt2*, *Cited2*), NTDs can be prevented by FA supplementation [85,86,87], in “curly tail” (*Grhl3*) mutant models, FA fails to induce such an effect [88,89].

Finally, studies have also demonstrated that a reduction in folate in diet increases the incidence of exencephaly in *Grhl3* models [90]. This brought into focus some other dietary supplements which are possibly needed in such cases [91], including the Mediterranean diet with a low consumption of meat products and high-fat/high-processed foods [92,93,94].

### 4.2. Inositol

Inositol is an alcohol often present in vitamin supplements and is involved in many cellular processes, mostly in various forms of second messengers. As such, studies on *Grhl3* mutant mice have shown that inositol deficiency increases the susceptibility to neural tube defects. At the same time, increasing the myo-inositol concentration normalized the closure of posterior NT in these embryos [95,96]. 

In medical application, inositol is known as a supplement efficient in preventing gestational diabetes [97,98,99]. Because there exist only a few studies on the efficacy of inositol in preventing NTDs or decreasing their incidence [100,101,102], including small numbers of participants, it remains elusive whether inositol alone, or in combination with FA, can further reduce the prevalence of NTDs [103,104]. Therefore, additional research into the potential mechanisms and roles of inositol during neural tube development, including its potential therapeutic use, is necessary [105].

## 5. Preventative, Management and Treatment Approaches

### 5.1. In Utero

Historically, the management of NTDs was usually conducted after birth. As such, it was of crucial importance to recognize the defect during pregnancy so that a safe delivery with a multidisciplinary team could be planned in advance. Even though cesarean sections are a controversial topic when talking about the delivery of children with NTDs, a variety of studies have shown better long-term motor function in children born via cesarean delivery [106].

Consequently, other approaches in the management of NTDs appeared. Because most NTDs could be diagnosed with an ultrasound in utero, it is important to examine the effects of prenatal surgery. Even though its benefits were questioned, results obtained through the randomized multicenter Management of Myelomeningocele Study (MOMS trial) were promising [107]. The MOMS trial has shown that performing prenatal surgery before the 26th week of gestation, in cases of myelomeningocele, decreased the risk of the neonate’s death or even the need for shunting by the age of 12 months. Furthermore, the mental and motor functioning score measurements indicated significant improvements at 30 months of age. On top of this, a variety of secondary outcomes which were improved with prenatal surgery were noticed: reduced degree of hindbrain herniation (Chiari II malformation), improved motor function and increased likelihood of being able to walk when compared to postnatal surgery [108].

With the discovery of many restorative effects of stem cells (SC), they have emerged as a potential therapeutic modality for NTDs in recent years. This is supported by a multitude of studies performed on animal models of NTDs, most of which included the induction of spinal open neural tube defects. As such, Sim et al. demonstrated significant changes in the NT re-closure capacity in chick embryos following stem cell transplantation [109]. Furthermore, Lee et al. reported that intra-amniotic transplantation of human embryonic stem cells into chick embryos following an NT incision enhanced its re-closure capacity, with a significantly shorter mean length of the ONTD observed in the treatment group [110]. Similar experiments were performed on fetal lambs with experimental myelomeningocele (MMC). Following prenatal neural stem cell (NSC) transplantation to the spinal cord, the results have shown a higher survival rate and an improvement in the walking ability of the treatment group [111]. Although clinical application of stem cells has shown a multitude of beneficial effects, the potential adverse effects as well as ethical issues related to their use still raises concerns in the field of regenerative medicine. These have been extensively covered by Volarevic et al. [112]. Due to their potential for unwanted differentiation, the ability to suppress the anti-tumor immune response and generate new blood vessels, mesenchymal stem cells (MSCs) could potentially promote tumor growth and metastasis. On the other hand, the use of human embryonic stem cells (hESCs) brings forth a multitude of ethical challenges, mainly related to the dilemma involving the destruction of a human embryo. On top of this, as hESCs are pluripotent cells, they are difficult to control after in vivo transplantation, potentially facilitating the development of teratomas [112]. Even though they are still seen as “morally superior“ to hESCs, ethical issues regarding the use of induced pluripotent stem cells (iPSCs) stem from their unlimited differentiation potential [112]. This brings forth concerns regarding their use in human cloning, as well as the generation of human embryos and human–animal chimeras. Even though stem cell therapies show great promise, the ethical challenges they pose warrant further consideration of the ethical implications and adaptation of the existing approach in order to facilitate their broad application.

### 5.2. Infant Care

Besides prenatal intervention and surgery, the treatment and management of NTDs can also be achieved postnatally. However, following the aforementioned MOMS trial, which has shown that in utero operations achieve better postoperative results, postnatal surgery no longer represents the most viable option. As such, more research is being conducted on alternatives, or novel combinations with postnatal surgery, that could yield more promising results. One such study is the one performed on 17 patients with myelomeningocele, aged between 1 month and 4 years, by Gupta et al. [113]. It included performing autologous stem cell transplantation into the spinal cord and the caudal space during or after postnatal surgery. Even though the results obtained are promising, more research is needed to confirm the significance and longevity of the observed benefits.

Additionally, because induced pluripotent stem cells (iPSCs) are reprogrammed somatic cells which become pluripotent, they can restore or reconstruct an entire organ or tissue, including its function. As such, an ongoing hypothesis in the field exists that neural crest cells could be reprogramed and transplanted in order to assist in the proper closure of the neural tube [114]. Positive effects of stem cells might even be improved by genetic modification [115] or by innovative ways of transplantation [116].

### 5.3. Adult Care

Because the management of a majority of NTDs occurs during the perinatal period, this means that the persistence of such lesions into adulthood is very uncommon. Moreover, it can be concluded that only the undiagnosed NTDs, which presented without obvious or with minimal symptoms, as well as the NTDs that could not be treated adequately at the time of the diagnosis, may be present in adults. Because the symptoms of untreated NTDs could worsen with time, especially after trauma, it is important to emphasize the options available for adult care with respect to their symptoms. As such, the symptoms that may be present since birth, and throughout adulthood, are lower extremity weakness as well as bowel and urinary overflow incontinence due to urinary retention. Despite the lack of treatment at an early age, there is a significant number of patients who underwent the operation as adults in order to manage their symptoms.

Godzik et al. published a case report about the treatment of an older (62 years old) patient who, after her symptoms worsened, underwent surgery [117]. Here, myelomeningocele presented with ulcerated lesions and CSF leakage, leading to an increase in the size of her ventricles. In order to stop the CSF leakage, which was present for 8 years prior to the surgery, the operation included the placement of an external ventricular drain. The whole procedure was successful, backpain symptoms were alleviated and the CSF fluid leakage was stopped.

## 6. Discussion

Neural tube defects (NTDs) are multifactorial disorders of the central nervous system arising during embryogenesis. They are a result of the failure of the morphogenetic process of the neural tube closure. Two of the most common types of NTDs are spina bifida and anencephaly. Despite numerous preventative approaches, NTDs represent, together with congenital heart defects, some of the most common serious birth defects worldwide [2].

**Figure 2:**
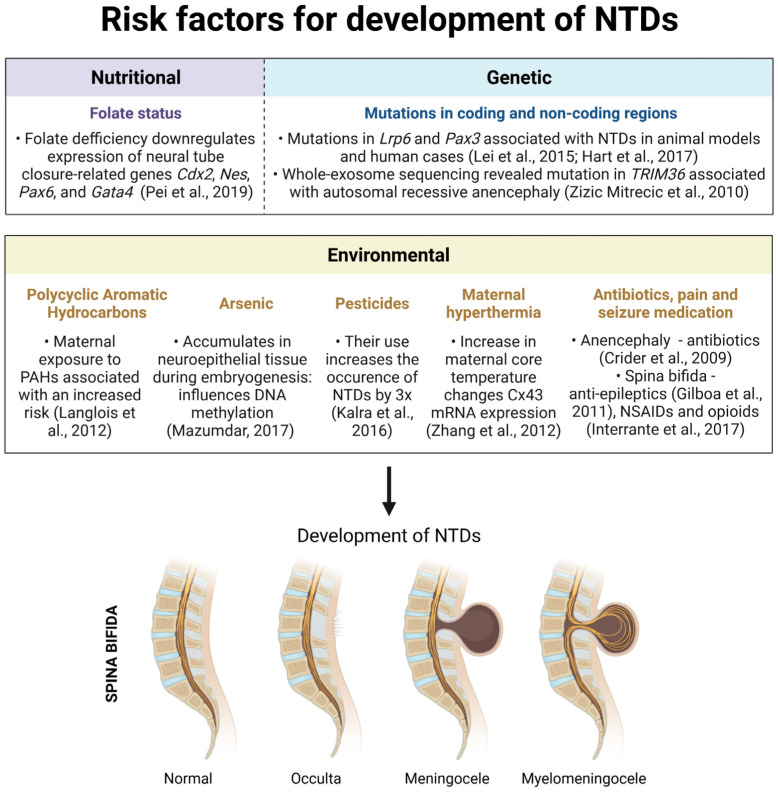
Summary of risk factors leading to development of NTDs. Created with www.BioRender.com (accessed on 26 March 2022).

With an increasing interest in the study and prevention of NTD development, several key risk factors, including nutritional, genetic and environmental, have been outlined throughout the years (Figure 2).

Because folate coenzymes are required for the synthesis of thymidylate and purines, their deficiency influences DNA methylation and induces histone modifications. As such, folates represent one of the crucial risk factors associated with the development of NTDs. On top of folate status, recent years have also seen a rise in studies demonstrating that low maternal serum vitamin B12 (cobalamin) levels increase the NTD risk to the developing embryo [118,119,120]. Although multiple studies have tried establishing a relationship between folate and cobalamin deficiency, results are still inconclusive, warranting further research [121]. Nevertheless, both of these represent essential water-soluble vitamins that regulate the cell metabolism, and their status within the organism is implicated in the development of NTDs.

On top of nutritional, there also exist multiple genetic factors associated with increased NTD risk. Generally speaking, most of research into the human NTDs etiology and associated treatment methods is currently being performed on animal models and supplemented with the next-generation sequencing of patient data. While murine models of NTDs have implicated the involvement of the *Lrp6* and *Pax3* genes in development of NTDs, WES studies identified mutations in *TRIM36* associated with the development of autosomal recessive anencephaly in an Indian family.

Even though genetic predispositions and the nutritional status of the mother play a role in the development of NTDs, arguably the biggest risk is posed by the gene–environment interactions, including PAHs, arsenic, pesticides, maternal hyperthermia and various drugs. Although the exact mechanism behind the role of PAHs in the development of NTDs remains elusive, studies suggest its role in decreasing the global DNA hydromethylation. By accumulating in the placenta, high PAH concentrations are associated with an increased risk of spina bifida. While PAHs mostly enter the organism through polluted air, arsenic is often consumed from underground water or contaminated food. By increasing the ROS production, inhibiting antioxidative enzymes and altering DNA methylation and repair mechanisms, arsenic has direct cytotoxic effects. Its teratogenic properties are connected with the damage of microvascular placental structures where it disrupts the transport of molecules and nutrition. Because arsenic also accumulates in the neuroepithelial tissue during embryogenesis, the genetic mutations and epigenetic changes it causes have been shown to directly influence NTD formation in both animal and human studies. On top of arsenic, another environmental pollutant directly related to NTD development is pesticide. Similar to arsenic, pesticides also have teratogenic and cancerogenic potential, leading to immunological and neurological dysfunction. The influence of pesticide exposure on the risk for NTDs is most seen in regions which rely on agriculture for development and, as such, utilize large quantities of pesticides. Multiple studies performed throughout the United States, Mexico and countries of Central and Southern Africa, where agricultural pesticides are commonly used, have demonstrated high rates of NTDs, at times even 2–3× higher than the average.

The development of NTDs is also related with the degree of hyperthermic damage and the overall exposure time. This includes an increase in maternal temperature by internal (virus) or external sources (sauna, hot tub and electric blanket). Even though the exact mechanisms of its action are still not fully elucidated, a study performed on golden hamsters suggested that hyperthermia influences *Cx34* gene expression. On the other hand, maternal body temperature can also be altered by viruses. Toxic metabolites produced during this process then pass through the placenta and directly affect the fetus. Both of these factors, internal and external, which lead to maternal hyperthermia, have been shown to increase the risk of the development of NTDs. Finally, NTDs as well as other congenital malformations can also be caused by the use of a variety of drugs, including antibiotics, pain and seizure medications. Despite all the positive effects of antibiotics, a multitude of studies have shown that they possess teratogenic properties, causing a variety of neurological disorders. The same holds true for seizure medications which have, despite their anti-epileptic effects, been implicated in causing spina bifida. While their consumption prior to pregnancy has not been related to NTD development in a significant manner as of yet, the ingestion of pain medication, including opioids and NSAIDs, during pregnancy, especially consecutively or simultaneously, is related to higher incidences of spina bifida and anencephaly when compared to singular use.

Even though NTDs are congenital malformations resulting from a complex interplay between the genes and the environment, a variety of preventative and management approaches have been defined throughout the last few decades, the most prominent of which includes folic acid food fortification. Regardless of the multitude of reported benefits, some studies reevaluating the mandatory FA fortification procedure are still present. One of the most prominent ones includes a population-based analysis of the dataset collected by the Food Fortification Initiative (FFI, Atlanta, GA, USA), a multi-sector partnership that works to reduce micronutrient deficiencies—including folic acid and iron—through the promotion of flour and rice fortification and aids with the planning and implementation of fortification programs. The subsequent analysis of the aforementioned dataset suggested that there exists only a weak correlation between NTD prevalence and the level of FA food fortification. Rather, the authors indicated that they observed a strong linear relationship between reduced NTDs and better socioeconomic status (SES) [122]. However, because Murphy et al. performed a population-level observational retrospective study, there exist several confounding factors potentially impacting the significance of their conclusion [123,124]. As such, the overarching consensus still remains, advocating for FA supplementation [125,126,127,128,129,130,131]. Nevertheless, as more data become available that could facilitate long-term studies, additional research is needed to further evaluate all the hypotheses and address their impact on public health.

Even though the health benefits of FA supplementation are well documented, risks associated with food fortification, potentially leading to high levels of unmetabolized folic acid (UMFA), are still being discussed [122,123,124,132,133,134]. A nested case–control study performed in the Boston Birth Cohort (BBC) suggested that higher concentrations of UMFA could be associated with the development of food allergies [132]. The authors postulate that this may be due to increased exposure to synthetic folic acid in utero or underlying genetic differences in synthetic folic acid metabolism. On the other hand, a prospective cohort study performed by Best et al. found no correlation with maternal late-pregnancy serum UMFA concentrations and infant allergic disease [135]. The study consisted of 561 mother–infant pairs in Western Australia and it measured serum UMFA as well as serum total folate. The infant allergic disease outcomes were taken to be medically diagnosed eczema, steroid-treated eczema, atopic eczema, IgE-mediated food allergy, allergen sensitization and medically diagnosed wheeze, all assessed at 1 year of age. Because this study was performed on a cohort of children at high risk of allergic disease, these results indicate that FA exposure in pregnancy is likely not an important factor contributing to the development of food allergies [136]. The results obtained by Best et al. are also mirrored in another prospective birth cohort study, including 1074 participants, performed by Molloy et al. [137]. It included measuring the red blood cell folate levels at 28–32 weeks gestation, followed by assessing food allergy outcomes in 1-year-old infants. While a majority of women involved in the study had high RBC folate levels, no association between third-trimester RBC folate and food allergies among the offspring was found.

On the other hand, a prospective clinical trial conducted on 30 healthy Brazilian adults, including 15 women, aged 27.7 y (95% CI: 26.4, 29.1 y), with a body mass index (in kg/m^2^) of 23.1 (95% CI: 22.0, 24.3), evaluated the effects of high-dose FA supplementation blood folate concentrations on the immune response [134]. The results indicated a ∼5-fold increase in serum folate concentrations after the intervention (*p* < 0.001), with an 11.9- and 5.9-fold increase in UMFA concentrations at days 45 and 90, respectively, when compared with the baseline (*p* < 0.001). Additional changes in the mRNA expression and the reduced number and cytotoxicity of NK cells were also observed, suggesting that higher levels of UMFA cause a weakened immune response [134].

Nevertheless, the results of many of these studies still provide discrepant results, with others indicating that plasma concentrations of UMFA arising from supplemental FA at a dose of 400 μg/d, in addition to FA from fortified foods, were low or undetectable in mothers and newborns, with a similar proportion of samples with detectable FA concentrations in both FA-treated and placebo groups [133]. An elaborate overview of FA and 5-MTHF supplementation as well as their association with UMFA syndrome can be found in Menezo et al. [138].

Because causes of folate deficiency which encourage implementation of food fortification strategies range from diet and lifestyle to pathological and pharmacological processes, it is also crucial to consider a personalized approach to supplementation. An increased understanding of how genetic variations affect responses to high-folate exposure might help address the concerns that were raised with respect to genetically susceptible subsets of the population [139,140]. In particular, some issues have been voiced with respect to those subsets of the population which carry mutations in the *DHFR* gene. A study by Orjuela et al. found that a subset of women with a homozygous 19 bp deletion in their *DHFR* gene (*DHFR19del*), and who took folic acid during pregnancy, were significantly more likely to have retinoblastoma in their offspring when compared with those with the *DHFR19del* alone [141]. Because this study concerned a common mutation found in 20% of the US population which slows conversion of UFA to intracellular folates [142], it is worth collecting more data in order to identify those groups with an increased risk of side effects following exposure to high levels of FA and adapt public health strategies accordingly. To the best of our knowledge, there is no other clinical data reporting the same results. Therefore, even though progress has been made in understanding folic acid’s impact on health, with a multitude of benefits being outlined throughout the years, some gaps in the knowledge still exist. These will have to be addressed in order to guide decisions on the most optimal approaches toward implementing population-wide food fortification strategies.

Considering that folic acid is an artificial precursor which needs to be reduced to dihydrofolate and tetrahydrofolate, and in order to investigate alternatives to FA food fortification, some studies proposed maternal supplementation with 5-methyltetrahydrofolate (5-MTHF) [95,96,97]. This theory is supported by a recently performed longitudinal study on 146 Japanese pregnant women who participated in the Chiba study of Mother and Child Health (C-MACH) [143]. The results suggested that 5-MTHF is actively transported through placental transporters to the fetus, suggesting its ability to maintain the folate status during pregnancy. Similar results were observed in a randomized controlled trial performed by Henderson et al. wherein the authors have shown that the L-5-MTHF group had higher RBC folate (1951 ± 496 nmol/L; *p* = 0.003) and plasma folate (52.0 nmol/L (42.7, 73.1 nmol/L); *p* = 0.023) levels at 12 weeks, when compared to the folic acid group (RBC folate, 1498 ± 580 nmol/L; plasma folate, 40.1 nmol/L (24.9, 52.7 nmol/L)). Because 5-MTHF does not accumulate in the blood in cases of reduced hepatic metabolism due to genetic variants or pharmacotherapies [144], unlike FA, it presents a viable alternative, avoiding some of the risks potentially associated with FA supplementation. However, large studies which would compare the efficacy of FA and 5-methyltetrahydrofolate are still lacking.

On top of the supplementation of maternal diet with folic acid, recent years have seen a rise in suggested benefits of inositol, an alcohol involved in many cellular processes. Nevertheless, if these preventative approaches have yielded little to no results, be it due to the improper following of predefined protocols or socio-environmental factors which make it hard to access these supplements, there also exist alternative management approaches in utero, in child and in adult care. As such, some of the most promising results can be observed in the MOMS trial, where prenatal surgery for cases of myelomeningocele was performed before the 26th week of gestation. This approach led to significant improvements in mental and motor functioning as well as decreasing the risk of the neonate’s death. Additional treatment approaches, mainly performed after birth, include stem cell transplantation during or after postnatal surgery. These have demonstrated positive results with increased neurological function observed in treated infants. Finally, even though the management and, in some cases, treatment of most NTDs takes place during the perinatal or early in the postnatal period, symptoms that could not be treated adequately at the time persist into adulthood. Some of these can be managed with surgical approaches, leading, in most cases, to the alleviation of symptoms.

Because NTDs are, together with congenital heart disease, congenital anomalies which appear in 6% of infants worldwide, they represent a large health, social and financial burden for the patient and society as a whole. However, recent advances in next-generation sequencing allows for deeper exploration of the NTDs etiology and provides hope that genetic underpinnings behind these disorders will be elucidated soon, facilitating novel prevention and management approaches.

## 7. Future Perspectives

### 7.1. Neural Organoid Systems

Despite many decades of research into the NTDs etiology, the mechanisms responsible for the impairment of neural tube development still remain elusive. One of the main problems is the lack of appropriate and reproducible models in which early developmental events could be studied [8,145]. Fortunately, these problems are beginning to be addressed through recent advancements on in vitro models of human tissue in the form of neural tube organoids [146,147,148,149]. As much as animal models have been successful in pinpointing some candidate genes, NT formation presents itself with clear differences among model species, including the number of initiating closure sites, the timings and the sequence of the events. All this is further fortifying the idea that more elaborate studies should be performed on human cells [8].

Most of the recent in vitro 3D cell models and organoids for studying the etiology and pathogenesis of NTDs include human pluripotent stem cells (hPSCs), human embryonic stem cells (hESCs) or, in some cases, murine ESCs [150,151,152,153,154]. Due to the stem cells’ innate ability to self-organize on extracellular matrices upon administration of exogenous signaling factors, they can adequately maintain both the dorsal–ventral organization [148] and rostro-caudal pattern [147]. These properties are similar to those of human neural tube development in vivo. Additionally, the utilization of murine ESCs for the formation of NT organoids is also not without its merits. Upon being genetically modified, murine ESCs can facilitate proof of principle studies on specific gene candidates which have, otherwise, been implied in the development of NTDs [8].

Bonnard et al. utilized patient-derived cerebral organoids to investigate the etiology and pathogenesis of NTDs in a family with recurrent anencephalic fetuses and recessive germline 21-bp in-frame deletion in *NUAK2* segregating with the disease [150]. The obtained results have shown that the loss of NUAK2 activity leads to a decreased Hippo signaling via cytoplasmic YAP retention, with apical colocalization of endogenous NUAK2 within the actomyosin network. Because this led to impaired nucleokinesis and apical constriction, the authors suggested that the NUAK2-Hippo signaling axis regulates cytoskeletal processes that govern cell shape during neural tube closure [150]. This interest in investigating gene candidates implied in the development of NTDs is also mirrored in a study performed by Cao et al. who aimed to elucidate the association between specific DNA damage response (DDR) genes and the risk of human NTD development [152]. Whole-genome sequencing and targeted sequencing identified a significant enrichment of rare deleterious *RAD9B* variants in spina bifida cases compared to controls. After establishing hESCs-derived neural organoids, the results have shown that *RAD9B* deficiency impeded their in vitro formation through the deregulation of cell adhesion genes, implicating this DDR gene as an NTD risk factor in humans [152].

Nevertheless, organoids still represent a relatively new approach for studying the NTDs etiology and pathogenesis. In order to investigate their viability as 3D models for human CNS development, Roll et al. have devised a study to quantify the spatiotemporal expression pattern and distribution of LewisX (LeX) trisaccharide motif and sulfation-dependent DSD-1 chondroitin sulfate glycosaminoglycan epitope in hiPSC-derived cerebral organoids [151]. The obtained results demonstrated distinct expression patterns of the glycoepithopes, as well as their potential carriers Protein tyrosine phosphatase receptor type Z1 (PTPRZ1) and Tenascin C (TNC), associated with Nestin-positive cells within rosette-like structures, which resemble the neural tube in vitro.

One of the limitations common to most classical cerebral organoids is the reduced complexity with regard to non-neural cell types [151]. Furthermore, human spinal-cord-like tissues induced from hPSCs are often insufficiently mature and lack some of the morphological features of neurulation. In order to address these limitations, Lee et al. have reported a novel protocol for establishing a model of human spinal-cord-like organoids (hSCOs) which exhibit neurulation-like tube-forming morphogenesis, synaptic network activity and cell differentiation into spinal-cord neurons and glial cells [154]. Although hSCOs represent great advancements in the field by replicating some of the features of neural tube formation in vivo, some limitations within the model can still be recognized. While the authors observed wedge-like cells emerging at the base of the folding NE layer undergoing tube formation, these cells did not exhibit a floor plate marker, indicating the absence of authentic medial hinge point (MHP) and dorsolateral hinge point (DLHP) cells. Additionally, no neural crest linear induction or dorsoventral patterning were observed [154]. On the other hand, Libby et al. have reported an organoid model of neural tube extension derived from hPSC aggregates caudalized with Wnt agonism [153]. Because these types of organoids can stimulate singular axial extension while maintaining multiple cell lineages, they can, quite faithfully, mirror various morphological and temporal gene expression patterns of NT development [155].

### 7.2. Surgical Approaches

Even though the surgical approach toward NTDs management has been rarely applied, there still exist cases in which it is of great help. The philosophy of open neural tube defect repair has, traditionally, been to preserve function at any cost, which meant an increased likelihood of future tethering of the spinal cord. On top of this, patients were often left with neural tissue injury, inclusion dermoid cysts, neurogenic bladder and bowel disfunction as well as a high-pressure bladder. A high-pressure bladder then predisposes them to upstream kidney damage, without any significant benefits of normal bladder function [156]. In order to mitigate this problem, novel surgical paradigms are being proposed, where some of the problems with the current standard of care are being addressed [156]. After performing postnatal surgical treatment of eight newborn infants with ONTD, which included the resection of the non-functional portion of the neural placode while performing direct nerve root stimulation, Eibach et al. have followed the progress of these patients with post-operative neuro-urological status and magnetic resonance imaging (MRI) at 3 weeks, 6 months and 2 years post-surgery. No worsening of the neurological status was observed following surgical intervention. Even though all patients exhibited neurogenic bladder and bowel dysfunction, there was no instance of a high-pressure bladder or inclusion dermoid cysts, suggesting a valid and viable alternative to traditional surgical approaches.

### 7.3. Engineered Nanomaterials and Teratogenicity

Finally, on top of developing novel approaches for the prevention and treatment of NTDs, it is also crucial to continue studying potential teratogens contributing to their development, one of which is engineered nanomaterials (ENMs) [157,158,159], including the silver nanoparticles (AgNPs) found in 75% of nanomedical products. Because they can cross the blood–brain and placental barriers, they present a hazard for the developing nervous system. Their teratogenic properties have been demonstrated on murine neural stem cells (mNSCs) where they were shown to exhibit significant toxicity [160]. Nevertheless, their harmful effects could be mitigated utilizing different coating agents for their surface stabilization. Additional studies performed on human embryonic stem cell-derived neural progenitors during neuronal differentiation have also revealed that AgNPs induce oxidative stress and inhibit neurogenesis through interfering with metal homeostasis and cholesterol biosynthesis [161]. These results mirrored earlier research performed on pregnant rat dams where repeated oral doses of AgNPs during pregnancy caused oxidative stress in hepatic tissues at ≥100 mg/kg/day and developmental toxicity at ≥1000 mg/kg/day [162]. Moreover, further studies on maternal intravenous injection of AgNPs in murine models demonstrated an increased accumulation of silver in the endometrium and the visceral yolk sac, with some even reaching embryos [163,164]. Because they are found in a variety of consumer and health products and, as such, may enter our bodies and cause a variety of health-related problems, further research into AgNPs and other ENMs properties and their role in embryo–fetal development is crucial and welcomed.

## Data Availability

Not applicable.
